# Polyethylene glycol 35 preserves endothelial barrier integrity by stabilizing the glycocalyx and intercellular junctions

**DOI:** 10.1042/BSR20260686

**Published:** 2026-11-11

**Authors:** Andrea Llaves-López, Queralt Barnat-Cabrera, Paula Manso-Álvarez, Joana Ferrer-Fàbrega, Carme Casal, Emma Folch-Puy

**Affiliations:** 1 Department of Experimental Pathology, Institut d'Investigacions Biomèdiques de Barcelona (IIBB), CSIC, Barcelona, Spain; 2 Institut d'Investigacions Biomèdiques August Pi i Sunyer (IDIBAPS), Barcelona, Spain; 3 Doctoral programme in Biomedicine, Universitat de Barcelona (UB), Barcelona, Spain; 4 Hepatobiliopancreatic Surgery and Liver and Pancreatic Transplantation Unit, Department of Surgery, Institute Clínic of Digestive and Metabolic Diseases (ICMDiM), Hospital Clínic, Universitat de Barcelona, Barcelona, Spain; 5 Network for Biomedical Research in Hepatic and Digestive Diseases (CIBEREHD), Barcelona, Spain; 6 Microscopy Unit, Institut d'Investigacions Biomèdiques de Barcelona (IIBB), CSIC, Barcelona, Spain

**Keywords:** adherens junction, endothelial barrier, endothelial glycocalyx, polyethylene glycols, tight junctions, vascular integrity

## Abstract

Endothelial dysfunction is a hallmark of numerous inflammatory and ischemic conditions, characterized by increased permeability, disruption of intercellular junctions and endothelial glycocalyx (eGCX) degradation. In the present study, we investigated the ability of polyethylene glycol 35 (PEG35) to preserve endothelial barrier integrity in cultured human umbilical vein endothelial cells (HUVECs) exposed to inflammatory, thrombotic, hypoxia-mimetic, and eGCX-disrupting stimuli. PEG35 mitigated the loss of cellular metabolic activity induced by apoptotic and hypoxia-mimetic stress. Functionally, PEG35 attenuated stimulus-induced increases in FITC-dextran permeability and helped maintain *trans*-endothelial electrical resistance (TEER) under pro-inflammatory and eGCX-disrupting agents. PEG35 also reduced LDH release and maintained higher TEER values in endothelial cells exposed to plasma collected from pancreas transplant recipients after reperfusion, which contained increased circulating levels of the eGCX component syndecan-1. Mechanistically, PEG35 reduced NF-κB p65 nuclear translocation under thrombin, tumor necrosis factor α, and hyaluronidase stimulation and attenuated inflammatory and endothelial activation-related transcriptional responses. PEG35 also modulated intercellular junction gene expression in a stimulus and target-dependent manner, helped maintain occludin and claudin-5 protein expression under selected injurious conditions, and limited the loss of eGCX-associated syndecan-1 and heparan sulfate staining after enzymatic degradation. Collectively, these findings support a multifactorial endothelial-protective effect of PEG35 involving preservation of the endothelial surface layer, maintenance of junctional integrity, and attenuation of inflammatory signalling. These results identify PEG35 as a potential vasoprotective candidate for conditions involving acute endothelial barrier injury.

## Background

The endothelial barrier, formed by a monolayer of endothelial cells lining blood vessels, plays a critical role in maintaining vascular homeostasis by regulating the exchange of fluids, solutes, and cells [1]. Its integrity is tightly regulated by the endothelial glycocalyx (eGCX) and intercellular junctions. The eGCX is a carbohydrate-rich matrix composed of proteoglycans, glycosaminoglycans (GAGs), and glycoproteins [2]. Proteoglycans, such as syndecans and glypicans, are anchored to the cell membrane and carry sulfated GAGs, including heparan sulfate (HS), chondroitin sulfate, dermatan sulfate, and keratan sulfate, while hyaluronan, a non-sulfated GAG, contributes to the gel-like structure by retaining water molecules and other GAGs [3]. Glycoproteins like selectins, integrins, and immunoglobulins mediate leukocyte recruitment and adhesion to activated platelets and endothelial cells [4]. Additional components such as albumin and thrombomodulin also integrate into the eGCX and contribute to vascular homeostasis [5].

In healthy endothelium, the eGCX undergoes continuous remodeling, balancing synthesis and constitutive or shear stress-induced shedding [6]. Its disruption and the release of eGCX-derived components into the bloodstream represent an early event in endothelial dysfunction, reported in conditions such as sepsis [3], cardiovascular disease [7], diabetes [8], COVID-19 [9], and ischemia-reperfusion injury (IRI) [10]. Intercellular junctions, including tight junctions and adherens junctions, are essential for forming adhesive interactions between neighboring endothelial cells [11]. Tight junctions are composed of the transmembrane proteins named occludins, claudins, and junctional adhesion molecules. These proteins are anchored to the actin cytoskeleton through the scaffold protein zona occludens-1 (ZO-1), thereby regulating paracellular permeability [12]. Adherens junctions are primarily formed by vascular endothelial cadherin (VE-cadherin), which mediates interactions between adjacent cells and associates with catenins to link to the cytoskeleton. Evidence suggests that disruption of the eGCX exposes and destabilizes intercellular junctions, contributing to the loss of endothelial barrier function [13]. Thus, preserving both the eGCX and intercellular junctions is crucial for ensuring vascular integrity under pathological conditions.

Composed of repeating ethylene glycol units, polyethylene glycols (PEGs) are synthetic hydrophilic polymers widely used in biomedical and pharmaceutical applications because of their water solubility, chemical stability, low intrinsic reactivity, and generally favorable biocompatibility profile [14,15]. These properties explain their incorporation into a broad range of formulations, including pharmaceutical excipients, PEGylated therapeutics, nanocarriers, and other clinically approved products [16]. However, PEGs constitute a heterogeneous family of polymers, and their biological behavior is strongly influenced by molecular weight, polymer architecture, formulation, dose, route, and duration of administration. Although no universally accepted cut-off defines low- versus high-molecular-weight PEGs, PEGs above approximately 10 kDa are often discussed as high-molecular-weight PEGs in pharmacological and toxicological settings, particularly in the PEGylated biotherapeutics literature [17]. Accordingly, PEG35, a 35 kDa molecular weight PEG, is generally considered a high-molecular-weight PEG. This distinction is biologically relevant because increasing PEG molecular weight prolongs blood persistence, reduces urinary clearance, favors alternative elimination pathways such as biliary excretion, and modifies tissue distribution [17,18].

Beyond their pharmacokinetic properties, high-molecular-weight PEGs may also exert relevant biophysical effects. Experimental studies have shown that PEGs can stabilize biological membranes, reinforce endothelial and epithelial barrier properties, and enhance cellular resistance to damage. PEG15–20 (15–20 kDa PEG) protected human lung endothelial cells *in vitro* by promoting cytoskeletal reorganization and activating barrier-reinforcing pathways [19]. In animal models, PEG15–20 maintained intestinal epithelial integrity in gut-derived sepsis [20] and reduced barrier disruption after radiation injury [21]. The use of PEG35 in an experimental model of intestinal IRI prevented HS shedding, reduced vascular permeability and edema, and significantly improved survival [22]. Moreover, Lopez et al. reported that supplementation of preservation solution with PEG35 during static cold storage of liver grafts safeguarded the tissue against syndecan-1 and HS shedding [23].

Nevertheless, potential limitations of PEG use should be interpreted in a setting-dependent manner. Repeated administration of PEGylated proteins or high-molecular-weight PEGs has been associated with intracellular PEG accumulation and cytoplasmic vacuolation, particularly in macrophages and, under some conditions, renal tubular cells [24]. However, available evidence suggests that PEG-associated vacuolation is often considered an adaptive cellular response and is not consistently linked to impaired cell or organ function [17,25]. Immune-related effects have also been described. Repeated systemic administration of PEG-containing or PEGylated products may induce anti-PEG antibodies or boost pre-existing anti-PEG antibodies, potentially altering pharmacokinetics, accelerating clearance from the circulation, and reducing therapeutic efficacy [26]. In selected cases, anti-PEG antibodies may contribute to complement activation-related pseudoallergic reactions, whereas immediate IgE-mediated hypersensitivity has been described predominantly with PEG-containing excipients or bowel preparations [27,28].

Other adverse findings are mainly related to specific formulations, routes, or exposure patterns. Gastrointestinal intolerance has been reported primarily with oral PEG formulations used as osmotic laxatives or bowel preparations and typically includes diarrhea, loose stools, nausea, bloating, and abdominal discomfort [29]. Renal findings related to PEG use appear uncommon and setting-dependent. They include renal tubular vacuolation in preclinical studies with repeated high-dose PEGylated proteins [30] and a small, generally reversible risk of renal dysfunction or acute kidney injury after high-volume oral PEG bowel preparation, particularly in susceptible patients or in those concomitantly receiving non-steroidal anti-inflammatory drugs [31]. More exceptional case reports have described severe renal injury in the setting of extreme misuse of PEG3350 (a lower-molecular-weight PEG used as an osmotic laxative), or sporadic oxalate nephropathy cases temporally associated with PEG3350 use, whereas rare immune-mediated renal reactions involving PEG or polysorbate excipients have also been described [32–34]. Therefore, PEG molecular weight, formulation, route, and duration of administration are determinants of both biological activity and safety.

In the present study, we aimed to investigate the protective effects of PEG35 on endothelial barrier integrity using cultured endothelial cells, with particular emphasis on its ability to preserve both the eGCX and intercellular junctions.

## Methods

### Human endothelial cell line

Human umbilical vein endothelial cells (HUVECs, C2519A, pooled donors, batch no. 0000663523, Lonza, Basel, Switzerland) were cultured in endothelial cell growth medium-2 (EGM-2; BulletKit CC-3162, Lonza). According to the supplier’s certificate of analysis, donor sex was reported as unknown. EGM-2 was prepared according to the manufacturer's protocol. Briefly, the EBM-2 basal medium was supplemented with EGM-2 SingleQuots, a set of essential growth supplements for endothelial cells. The medium was further supplemented with 1% penicillin–streptomycin solution (P4333, Sigma–Aldrich, St. Louis, MO, U.S.A.), providing final concentrations of 100 U/ml penicillin and 100 μg/ml streptomycin. Cells were incubated at 37°C in a humidified atmosphere containing 5% CO_2_.

Replicate cultures were obtained by trypsinization and used for experiments between passages 2 and 7. Before experimental use, cultures were routinely inspected under an inverted phase-contrast microscope to verify cell attachment, monolayer formation, and cobblestone-like endothelial morphology. Endothelial phenotype was further supported by immunofluorescence staining for CD31.

### Morphological and immunophenotypic characterization of HUVECs

Before experimental use, HUVEC cultures were routinely examined by phase-contrast microscopy to verify the characteristic cobblestone-like morphology of confluent endothelial cells. Briefly, HUVECs were seeded in culture plates and maintained in complete endothelial growth medium until reaching confluence. Phase-contrast images were acquired using an inverted microscope (Olympus CKX53, Olympus, Shanghai, China) prior to treatment. Only cultures displaying homogeneous monolayer formation and typical cobblestone-like endothelial morphology were used for subsequent experiments. The representative image is shown in Supplementary Figure S1.

For CD31 immunofluorescence staining, HUVECs were seeded at a density of 2.5 × 10^4^ cells per well in 24-well plates and allowed to adhere for 24 h. Cells were washed with PBS (AM9625; Invitrogen, Massachusetts, U.S.A.) and fixed with a neutral buffered 10% formalin solution (HT501128, Merck KGaA, Darmstadt, Germany) for 15 min at room temperature. After washing with PBS, cells were incubated with 2% bovine serum albumin in PBS blocking solution for 30 min, followed by overnight incubation at 4°C with mouse monoclonal anti-CD31 [JC/70A] primary antibody (ab9498; Abcam, Cambridge, U.K.) at a concentration of 1 μg/ml in blocking solution. Cells were then washed with PBS and incubated for 2 h at room temperature in the dark with Alexa Fluor® 488-conjugated goat anti-mouse IgG H&L secondary antibody (ab150117, Abcam, Cambridge, U.K.) diluted 1:500 in blocking solution. After washing with PBS, nuclei were stained with DAPI (D9542; Sigma–Aldrich, St. Louis, MO, U.S.A.) at 0.2 μg/ml in Milli-Q water for 5 min at room temperature in the dark. Fluorescence imaging of cells was performed using an inverted fluorescence microscope (Olympus CKX53, Olympus, Shanghai, China). The representative image is shown in Supplementary Figure S1.

### Human blood samples

Human blood samples were collected from 38 pancreas transplantation recipients after obtaining informed consent, in accordance with protocols approved by the Institutional Review Board of Hospital Clínic Barcelona (No. HCB/2020/0499) and with the 1964 Helsinki Declaration and its later amendments [35].

Venous blood samples were obtained from pancreas transplant recipients 10 min after pancreatic graft reperfusion (PR plasma) using BD Vacutainer® PPT™ Plasma Preparation Tubes (5 ml, 13 × 100 mm; 362795; BD, Canada), which contain spray-dried K_2_EDTA anticoagulant and an inert gel separator. Samples were centrifuged at 1100×*g* for 11 min (Hettich EBA 270 centrifuge, Cultek, Spain), and plasma was subsequently collected and stored at −80°C until further analysis. Blood samples from healthy volunteers (*n* = 14) were collected and processed using the same tube type and centrifugation conditions. For the *in vitro* experiments, individual plasma samples from pancreas transplant recipients and healthy volunteer controls were used separately; no pooled plasma samples were used.

According to the institutional thrombosis prophylaxis protocol for pancreas transplantation, low-molecular-weight heparin is administered at 20 mg every 12 h starting 8 h after pancreas reperfusion [36]. Therefore, systemic heparin prophylaxis had not yet been initiated at the time of collection of the 10-min PR plasma samples used for the *in vitro* assays.

### PEG35 preparation

Polyethylene glycol 35 (PEG35) was obtained from IGL in crystalline solid form (100006, lot CHI190151, Lissieu, France). PEG35 was prepared as a 4% (w/v) solution in EBM-2 basal medium supplemented with 0.5% (v/v) fetal bovine serum (FBS). Briefly, PEG35 was dissolved in the culture medium under sterile conditions in a laminar-flow hood and gently mixed until complete dissolution. The PEG35-containing medium was then sterilized by passage through a 0.22 μm sterile syringe filter before being added to the cell cultures.

The 4% PEG35 concentration was selected based on previous dose-response studies from our group showing that this concentration is biologically active in inflammatory cell models. In AR42J pancreatic acinar cells, PEG35 concentrations ranging from 0.5% to 6% reduced inflammatory gene expression and cell death induced by cerulein, tumor necrosis factor α (TNFα), and staurosporine [37]. In differentiated THP-1 macrophages, PEG35 concentrations ranging from 1% to 6% attenuated IL1β induction triggered by acute pancreatitis-derived exosomes or LPS [38]. Based on these prior data, 4% PEG35 was selected as a biologically active concentration for the present endothelial cell experiments. Its compatibility with HUVECs under the experimental conditions used was assessed in the cell metabolic activity assay described below.

### Cell metabolic activity

HUVECs were seeded at a density of 1 × 10^4^ cells per well in 96-well culture plates and allowed to adhere for 48 h. Cells were then treated with 4% PEG35 diluted in culture medium for 30 min before exposure to the appropriate stimuli for 4 h: 200 and 250 nM staurosporine (S4400; Merck KgaA, Darmstadt, Germany) as an apoptotic inducer, or 100 and 150 μM cobalt chloride (CoCl_2_) (15862; Merck KgaA, Darmstadt, Germany), a commonly used hypoxia-mimetic agent.

In a separate set of experiments, cells were pre-treated with 4% PEG35 for 30 min and then exposed for 24 h to culture medium containing 5% (v/v) PR plasma, in the absence or presence of PEG35 according to the experimental condition. For PEG35-treated conditions, the polymer was maintained throughout the 24-h plasma exposure. Following the respective incubation periods, cell metabolic activity, used as an indirect readout of viable cells, was assessed using the CellTiter 96® Aqueous One Solution Cell Proliferation Assay (G3580; Promega, Madison, WI, U.S.A.), according to the manufacturer’s instructions. This assay is based on the MTS colorimetric method. Briefly, the culture medium was aspirated and replaced with a solution containing 20 μl of the One Solution reagent, comprising the MTS tetrazolium compound, and 100 μl of fresh culture medium. Plates were incubated for 1 h at 37°C in a humidified atmosphere containing 5% CO_2_. The MTS reagent is reduced by metabolically active cells to form a colored formazan product. The amount of formazan, measured by absorbance at 490 nm using an automated microplate reader (Thermo Scientific™ Multiskan SkyHigh Microplate Spectrophotometer; Thermo Scientific, Waltham, MA, U.S.A.) is proportional to the number of viable cells under the assay conditions. Results were expressed as percentages relative to the untreated control group.

For stimulus-based MTS assays, four independent experiments were performed, with each condition analyzed in technical triplicate. Technical triplicates were averaged to obtain one value per condition and independent experiment.

The plasma-exposure MTS assay was conducted across five experimental runs, each of which included untreated control and PEG35-alone conditions as internal plate controls, together with a subset of the plasma samples. Control and PEG35-alone conditions were analyzed in technical duplicate in each run, and duplicate values were averaged to obtain one value per condition and experimental run. PR plasma samples from 38 pancreas transplant recipients were individually analyzed in technical duplicate in the absence or presence of PEG35, and duplicate values were averaged before statistical analysis. Control and PEG35-alone conditions were matched by experimental run, whereas PR plasma and PEG35 + PR plasma conditions were matched by individual plasma samples.

### Endothelial cell permeability

Endothelial permeability across HUVEC monolayers was determined using 24-well plates and Transwell polycarbonate membrane cell culture inserts with a 0.4-μm pore size (Corning Inc., Corning, NY, U.S.A.). Briefly, HUVECs were seeded at a density of 5 × 10^4^ cells per insert and cultured for 48 h, or until the formation of a confluent and tight monolayer.

Cells were treated with 4% PEG35 diluted in a culture medium for 1 h prior to treatment with the appropriate stimuli for 2 h: 2 U/ml thrombin (T4393; Merck KgaA, Darmstadt, Germany), 50 ng/ml vascular endothelial growth factor (VEGF; 100-20; PeproTech, Cranbury, NJ, U.S.A.), 15 U/ml hyaluronidase (H1136; Merck KgaA, Darmstadt, Germany), 60 ng/ml TNFα (300-01A; PeproTech, Cranbury, NJ, U.S.A.), and 20 ng/ml interferon γ (IFNγ; 300-02; PeproTech, Cranbury, NJ, U.S.A.). The medium was then aspirated and replaced with fresh medium containing 1 mg/ml of 70-kDa fluorescein isothiocyanate (FITC)-dextran (46945; Merck KgaA, Darmstadt, Germany). Following a 1-h incubation, the content of the lower chamber was collected, and endothelial permeability was quantified by evaluating the translocation of FITC-dextran across the HUVEC monolayer.

Fluorescence intensity was measured using a SAFAS Xenius XOF fluorimeter (SAFAS Monaco, Monaco) equipped with excitation and emission monochromators rather than fixed optical filters. FITC-dextran fluorescence was measured using excitation and emission monochromator settings of 485 and 520 nm, respectively, with a spectral bandwidth of 10 nm. The concentration of FITC-dextran in each sample was determined using a standard calibration curve.

Six independent experiments were performed, with each condition analyzed in technical duplicate. Technical duplicates were averaged before statistical analysis, and each independent experiment was considered one biological replicate.

### *Trans*-endothelial electrical resistance

Endothelial barrier function was assessed by measuring *trans*-endothelial electrical resistance (TEER) using the Millicell ERS-3 Volt-Ohm meter (Merck KgaA, Darmstadt, Germany), following the manufacturer’s instructions. Briefly, endothelial cells were seeded onto Transwell polycarbonate membrane cell culture inserts (0.4-μm pore size) and cultured for 48 h, or until a confluent and tight monolayer was formed.

Cells were treated with 4% PEG35 diluted in culture medium for 1 h prior to incubation with one of the following stimuli for 2 h: 2 U/ml thrombin, 50 ng/ml VEGF, 15 U/ml hyaluronidase, 60 ng/ml TNFα, or 20 ng/ml IFNγ. In a separate set of experiments, cells were pre-treated with 4% PEG35 for 30 min and then exposed for 2 h to culture medium containing 5% (v/v) PR plasma, in the absence or presence of PEG35 according to the experimental condition. For PEG35-treated conditions, the polymer was maintained throughout the 2-h plasma exposure.

Following treatment, TEER was recorded by immersing both ends of the electrode probe, placing the shorter tip in the apical chamber of the insert and the longer tip in the basolateral chamber of the outer well. For experimental conditions using PEG35-supplemented medium, TEER values from cell-free inserts containing PEG35 were used as blanks. For conditions using standard medium, blanks consisted of inserts without cells and without PEG35. Untreated cells served as controls. TEER values were calculated using the formula: [average resistance of experimental wells − blank] × membrane area (0.33 cm^2^), yielding final results expressed in Ω·cm^2^. Higher TEER values indicate greater endothelial barrier integrity, reflecting tighter intercellular contacts and reduced paracellular permeability.

For stimulus-based TEER assays, five independent experiments were performed, with each condition analyzed in technical duplicate. Technical duplicates were averaged to obtain one value per condition and independent experiment.

The plasma-exposure TEER assay was conducted across five experimental runs, each of which included untreated control and PEG35-alone conditions as internal plate controls, together with a subset of the plasma samples. Control and PEG35-alone conditions were analyzed in technical duplicate in each run, and duplicate values were averaged to obtain one value per condition and experimental run. PR plasma samples from 38 pancreas transplant recipients were individually analyzed in technical duplicate in the absence or presence of PEG35, and duplicate values were averaged before statistical analysis. Control and PEG35-alone conditions were matched by experimental run, whereas PR plasma and PEG35 + PR plasma conditions were matched by individual plasma samples.

### Lactate dehydrogenase activity

HUVECs were seeded at a density of 1 × 10^4^ cells per well in 96-well culture plates and allowed to adhere for 24–48 h or until confluence. Cells were pre-treated with 4% PEG35 for 30 min and then exposed for 24 h to culture medium containing 5% (v/v) PR plasma or 5% (v/v) plasma from healthy controls, in the absence or presence of PEG35, according to the experimental condition. For PEG35-treated conditions, the polymer was maintained throughout the 24-h plasma exposure.

Following incubation, cell culture supernatants were collected, and lactate dehydrogenase (LDH) activity was measured as an indicator of cytotoxicity using the CytoTox 96® Non-Radioactive Cytotoxicity Assay (G1780; Promega; Madison, WI, U.S.A.), according to the manufacturer’s instructions. Briefly, LDH released into the culture supernatants was quantified by providing lactate, NAD^+^, and tetrazolium salt as substrates in the presence of diaphorase, resulting in the conversion of tetrazolium salt into a red formazan product. The intensity of the color is proportional to the amount of LDH released. Absorbance was measured at 490 nm using an automated microplate reader (Multiskan SkyHigh Microplate spectrophotometer; Thermo Scientific™, Waltham, MA, U.S.A.). LDH activity in culture supernatants was corrected by subtracting baseline LDH activity present in the corresponding plasma-containing medium and expressed in mU/ml.

The LDH assay was conducted across five experimental runs, each of which included untreated control and PEG35-alone conditions as internal plate controls, together with a subset of the plasma samples. Control and PEG35-alone conditions were analyzed in technical duplicate in each run, and duplicate values were averaged to obtain one value per condition and experimental run. Individual plasma samples from 14 healthy controls and 38 pancreas transplant recipients were analyzed in technical duplicate, with PR plasma samples assessed in the absence or presence of PEG35. Duplicate values were averaged before statistical analysis. Control and PEG35-alone conditions were matched by experimental run, healthy control plasma and PR plasma samples represented independent groups, and PR plasma and PEG35 + PR plasma conditions were matched by individual plasma sample.

### Enzyme-linked immunosorbent assay

Concentrations of eGCX degradation product syndecan-1 were quantified in human blood samples collected from pancreas transplantation recipients and healthy controls using enzyme-linked immunosorbent assay (ELISA) and following the manufacturer’s instructions (136 950.640.192; Diaclone, France). Plasma samples from 38 pancreas transplant recipients and 14 healthy controls were analyzed. Each individual plasma sample was measured in technical duplicate in a single ELISA assay, and duplicate values were averaged before statistical analysis. Each individual plasma sample was considered one biological replicate.

### Immunofluorescence

To assess the activation and nuclear translocation of the transcription factor nuclear factor kappa B (NFκB), HUVECs were seeded at a density of 1.2 × 10^4^ cells per well in 48-well plates and allowed to adhere for 48 h. Cells were then treated with 4% PEG35 diluted in culture medium for 30 min prior to incubation with one of the following stimuli for 2 h: 2 U/ml thrombin, 15 U/ml hyaluronidase, or 60 ng/ml TNFα.

Cells were fixed with neutral buffered 10% formalin solution for 15 min at room temperature, followed by a 2-min permeabilization with Triton X-100 (T8787; Sigma–Aldrich, St. Louis, MO, U.S.A.). Immunostaining was performed by overnight incubation with a mouse monoclonal anti-NFκB p65 (F6) primary antibody from Santa Cruz (sc-8008, 1:400 dilution; Santa Cruz Biotechnology, CA, U.S.A.). Alexa Fluor® 488-conjugated goat anti-mouse IgG H&L (preadsorbed; ab150117, Abcam, Cambridge, U.K.) was used as the secondary antibody. Following a 2-h antibody incubation, cells were washed with PBS, and nuclei were counterstained with DAPI (0.2 μg/ml in Milli-Q water) for 5 min at room temperature in the dark. Cells were then washed with PBS, and NFκB p65 subunit localization was examined using the inverted fluorescence microscope Olympus CKX53 (Olympus, Shanghai, China).

NF-κB p65 nuclear translocation was quantified from immunofluorescence images. For each experimental condition, five randomly selected fields of comparable size were analyzed from each independent experiment. Image analysis was performed blinded to the experimental condition. Nuclei were identified using the DAPI channel, and cells were classified as positive for nuclear p65 translocation when p65 staining clearly overlapped with the DAPI-positive nuclear area. For each field, the percentage of cells showing nuclear p65 translocation was calculated as the number of nuclei with nuclear p65 staining divided by the total number of DAPI-positive nuclei. Field-level percentages were averaged to obtain one value per condition for each independent experiment.

Three independent experiments were performed, with each condition analyzed in technical duplicate. Technical duplicates were averaged before statistical analysis, and each independent experiment was considered one biological replicate.

### Quantitative real-time PCR

HUVECs were seeded at a density of 9 × 10^4^ cells per well in 12-well culture plates and allowed to adhere for 24 h. Cells were then treated with 4% PEG35 diluted in culture medium for 30 min before stimulation for 3 h with one of the following agents: 2 U/ml thrombin, 60 ng/ml TNFα, and 15 U/ml hyaluronidase. After the incubation period, supernatants were removed and total RNA was extracted using Nucleozol (740404.200; Macherey-Nagel, Düren, Germany) according to the manufacturer’s instructions. RNA was purified by isopropanol precipitation and ethanol washing, then resuspended in RNAse-free water.

RNA concentration was measured using a Nanodrop 1000 spectrophotometer (Thermo Scientific, Waltham, MA, U.S.A.), and RNA quality was assessed by evaluating A260/A280 and A260/A230 absorbance ratios. RNA samples were stored at −80°C until further analysis. Reverse transcription was performed using the iScript™ gDNA Clear cDNA Synthesis Kit (172-5035; Bio-Rad Laboratories, Hercules, CA, U.S.A.) following the manufacturer’s indications.

PCR amplification was carried out using the SsoAdvanced™ Universal SYBR® Green Supermix (172-5270; Bio-Rad Laboratories, Hercules, CA, U.S.A.) on a CFX Real-Time PCR Detection System (Bio-Rad Laboratories, Hercules, CA, U.S.A.). Each reaction was performed in a final volume of 10 μl containing cDNA corresponding to 50 ng of input RNA and 450 nM of the corresponding forward and reverse primers. A no-template control was included for each gene. All primers were custom-designed from Proquinorte (Zamudio, Bilbao, Spain), and their sequences and target genes are listed in Table 1. The specificity of the amplicons was confirmed by melting curve analysis. All reactions were performed in technical duplicate, and threshold cycle (Ct) values were normalized to GAPDH expression using the ΔCt method [39]. Data are presented as relative expression values normalized to untreated control cells.

**Table 1:** Primers used for quantitative real-time PCR analysis

PRIMER	SEQUENCE (5′−3′)
***GAPDH***	Fwd: TCGGAGTCAACGGATTTG
	Rev: CAACAATATCCACTTTACCAGAG
***Occludin***	Fwd: GGACTGGATCAGGGAATATC
	Rev: ATTCTTTATCCAAACGGGAG
***ZO-1***	Fwd: TTGTCTTCAAAAACTCCCAC
	Rev: GACTCACAGGAATAGCTTTAG
***VE-cadherin***	Fwd: CGCAATAGACAAGGACATAAC
	Rev: TATCGTGATTATCCGTGAGG
***Claudin-5***	Fwd: TCTTTACTCCATCGGCAG
	Rev: TTTTGGAGAGAGTTCAAACC
***IL1β***	Fwd: CTAAACAGATGAAGTGCTCC
	Rev: GGTCATTCTCCTGGAAGG
***IL6***	Fwd: GCAGAAAAAGGCAAAGAATC
	Rev: CTACATTTGCCGAAGAGC
***eNOS***	Fwd: CAACCCCAAGACCTACG
	Rev: CGCAGACAAACATGTGG
***ET-1***	Fwd: CAAGCAGGAAAAGAACTCAG
	Rev: CTGGTTTGTCTTAGGTGTTC

Five independent experiments were performed, with each condition analyzed in technical duplicate. Technical duplicates were averaged before statistical analysis, and each independent experiment was considered one biological replicate.

### Western blot

HUVECs were seeded at a density of 2 × 10^5^ cells per well in 6-well culture plates and allowed to adhere for 24 h. Cells were then treated with 4% PEG35 diluted in 0.5% FBS culture medium for 30 min prior to incubation with one of the following stimuli for 4 h: 2 U/ml thrombin, 60 ng/ml TNFα, and 15 U/ml hyaluronidase. After the incubation period, supernatants were removed, and total proteins from HUVECs were extracted using ice-cold RIPA buffer supplemented with a protease inhibitor cocktail (4693124001, Sigma–Aldrich, St. Louis, MO, U.S.A.). The lysates were centrifuged at 15,000×***g*** for 20 min at 4°C, and the supernatants were collected. Protein concentrations were determined using the Bradford protein assay (5000006; Bio-Rad Laboratories, Hercules, CA, U.S.A.).

SDS–PAGE was performed on 10% or 12% polyacrylamide gels, and proteins were subsequently transferred onto PVDF membranes (Bio-Rad, Hercules, CA, U.S.A.) using the Trans-Blot® Turbo™ Transfer System (1704150; Bio-Rad, Hercules, CA, U.S.A.). Membranes were blocked for 5 min in blocking buffer (12010020, Bio-Rad Laboratories, Hercules, CA, U.S.A.), followed by overnight incubation at 4°C with the corresponding primary antibodies: rabbit polyclonal anti-claudin-5 antibody (ab15016, 1:500 dilution, Abcam, Cambridge, U.K.), rabbit monoclonal anti-occludin antibody [EPR20992] (ab216327, 1:500 dilution; Abcam, Cambridge, U.K.), and mouse monoclonal anti-vinculin antibody (V4505; 1:5000 dilution, Sigma–Aldrich, St. Louis, MO, U.S.A.). Following incubation, blots were washed and incubated with corresponding HRP-conjugated secondary antibodies: goat anti-rabbit IgG (H+L) (170-6515, 1:2000 dilution, Bio-Rad Laboratories, Hercules, CA, U.S.A.) and goat anti-mouse IgG (H+L) (170-6516, 1:5000 dilution, Bio-Rad Laboratories, Hercules, CA, U.S.A.). Signal detection was performed using enhanced chemiluminescence reagents (1705061; Bio-Rad Laboratories, Hercules, CA, U.S.A.), and chemiluminescent signals were captured using the ChemiDoc™ Touch Imaging System (Bio-Rad Laboratories, Hercules, CA, U.S.A.). Protein expression levels were quantified and normalized to vinculin.

Five independent experiments were performed, and each independent experiment was considered one biological replicate.

### Immunofluorescence staining of eGCX components

HUVECs were seeded at a density of 2.5 × 10^4^ cells per well on poly-D-lysine (P4707; Merck KgaA, Darmstadt, Germany) coated cell slides and allowed to adhere for 24 h. The cells were then incubated with 4% PEG35 diluted in 0.5% FBS culture medium for 30 min prior to incubation with one of the following stimuli: 0.5 U/ml heparinase III (C3667; Sigma–Aldrich, St. Louis, MO, U.S.A.) for 2 h to induce HS shedding or with 0.5 μg/ml human recombinant matrix metalloproteinase 7 (MMP7; M4565, Sigma–Aldrich, St. Louis, MO, U.S.A.) for 1 h to induce syndecan-1 shedding.

After stimulation, cells were washed with PBS and fixed in a neutrally-buffered 10% formalin solution for 15 min. Permeabilization was omitted to preserve the integrity of the eGCX. After washing with PBS, the cells were incubated in blocking solution for 30 min and subsequently incubated with the appropriate primary antibody overnight at 4°C. For HS labeling, a rat monoclonal anti-HS antibody (ab2501; Abcam, Cambridge, U.K.) was used at a 1:100 dilution. For syndecan-1 detection, a mouse monoclonal anti-syndecan-1 antibody (sc-12765; Santa Cruz Biotechnology, Texas, U.S.A.) was used at a 1:50 dilution. After primary antibody incubation, cells were washed with PBS and incubated with the corresponding secondary antibody for 2 h at room temperature in the dark: an Alexa Fluor® 488-conjugated goat anti-rat IgG H&L antibody (preadsorbed; ab150165, Abcam, Cambridge, U.K.) was used for HS detection, and an Alexa Fluor® 488-conjugated goat anti-mouse IgG H&L antibody (preadsorbed; ab150117; Abcam, Cambridge, U.K.) was used for syndecan-1 detection. After washing with PBS, nuclei were counterstained with DAPI (0.2 μg/ml in Milli-Q water) for 5 min at room temperature in the dark. Coverslips were then washed with PBS and mounted with ProLong™ Glass Antifade Mountant (P36984; Invitrogen, Massachusetts, U.S.A.).

Cells were imaged using an inverted Nikon Eclipse Ti2-E microscope (Nikon Instruments, Melville, NY, U.S.A.) equipped with an Andor Dragonfly spinning disk confocal unit. All imaging was conducted using a Plan Apo lambda 60× oil-immersion objective (numerical aperture 1.40). Samples were excited with 405 and 488 nm laser diodes. The laser beam was coupled into a multimode fiber and passed through the Andor Borealis unit, which reshaped the beam from a Gaussian profile into a uniform flat-top profile before transmission through a 40-μm pinhole disk. All images were acquired using a high-resolution scientific complementary metal oxide semiconductor camera (Zyla 4.2, 2.0 Andor, Oxford Instruments Company). Image acquisition was performed using Fusion software (Oxford Instruments), and image processing and analysis were conducted using Image J/Fiji open-source software.

Images were acquired as z-stacks using identical acquisition settings within each experiment. The z-step size was set according to Nyquist sampling criteria using the microscope acquisition software. Maximum-intensity projections were generated from the z-stacks using ImageJ/Fiji and used for subsequent quantification.

Quantification of HS and syndecan-1 staining was performed on the Alexa Fluor® 488 channel. Background was subtracted using the same parameters for all images within the same staining experiment, and a fixed threshold was applied to identify the specific immunostained area. The positively stained area was measured using ImageJ/Fiji and expressed as the percentage of immunostained area relative to the total image area. Field-level values were averaged to obtain one value per condition for each independent experiment, which was used for statistical analysis.

Three independent experiments were performed for eGCX immunofluorescence analyses, with each condition analyzed in technical duplicate. Technical replicates were averaged before statistical analysis. Each independent experiment was considered one biological replicate.

### Statistical analysis

Statistical analysis was conducted using GraphPad Prism 10.3.1 (GraphPad Software, Boston, MA, U.S.A.). Technical duplicates or triplicates were averaged before statistical analysis and were not treated as independent observations. For stimulus-based assays, each independent experiment was considered one biological replicate. For plasma-exposure assays, each individual plasma sample was considered one biological replicate, whereas untreated control and PEG35-alone conditions were represented by the corresponding independent experimental runs.

Quantitative data are shown as mean ± SEM and presented as scatter dot plots showing the individual biological or experimental replicates, as appropriate. Pairwise statistical tests were two-sided, and *P<*0.05 was considered statistically significant. When correction for multiple comparisons was applied, significance was determined using adjusted *P-*values.

For MTS assays evaluating the effects of PEG35 under staurosporine or CoCl_2_ stimulation, data were analyzed using ordinary two-way repeated-measures ANOVA, with stimulus concentration and PEG35 treatment as factors, followed by Sídák’s multiple-comparisons test.

For FITC-dextran permeability and TEER assays performed with thrombin, VEGF, hyaluronidase, TNFα, and IFNγ, data were analyzed using two-way repeated-measures ANOVA, with stimulus and PEG35 treatment as factors, followed by Šídák’s multiple-comparisons test. The same approach was applied to NFκB p65 nuclear translocation, qPCR analyses, and Western blot densitometry. Each measured outcome (including each individual gene and each protein) was analyzed separately. For qPCR analyses, statistical comparisons were performed on ΔCt values, whereas data are presented as relative expression values normalized to untreated control cells.

For plasma-exposure MTS and TEER assays, two prespecified matched comparisons were performed within each panel. Untreated control and PEG35-alone conditions were compared as matched observations across five experimental runs, whereas PR plasma and PEG35 + PR plasma conditions were matched by individual plasma samples. Comparisons were analyzed using a paired *t*-test when the paired differences were normally distributed or a Wilcoxon matched-pairs signed rank test when normality was not met. *P-*values were adjusted for the two prespecified comparisons within each panel using the Šídák method.

For the plasma-exposure LDH assay, three prespecified comparisons were performed. Untreated control and PEG35-alone conditions were compared across five experimental runs using a paired *t*-test after confirming the normality of the paired differences. Healthy control plasma and PR plasma samples were compared as independent groups using Welch’s unpaired *t*-test. PR plasma and PEG35 + PR plasma conditions were matched by individual plasma sample and compared using a paired *t*-test. *P*-values were adjusted for the three prespecified comparisons using the Šídák method.

For ELISA analysis of plasma syndecan-1 levels, healthy control plasma and PR plasma samples were compared using a two-sided Mann−Whitney test because the data did not follow a normal distribution.

For immunofluorescence quantification of eGCX components, field-level values were averaged within each technical replicate, and technical duplicate values were subsequently averaged to obtain one value per condition for each independent experiment. Syndecan-1 and HS staining were analyzed separately using repeated-measures one-way ANOVA, followed by Šídák’s multiple-comparisons test.

## Results

### PEG35 mitigated the loss of endothelial cell metabolic activity induced by apoptotic and hypoxia-mimetic stress

We first evaluated the effect of PEG35 on endothelial cell metabolic activity under stress conditions. MTS assay was performed after 4 h exposure to the apoptotic agent staurosporine (200 and 250 nM) or hypoxia-mimetic agent CoCl_2_ (100 and 150 μM). The 4% PEG35 concentration was selected based on prior dose-response studies from our group in inflammatory cell models, where this concentration showed biological activity without apparent cytotoxicity [37,38]. The concentrations of staurosporine and CoCl_2_ were selected based on preliminary optimization experiments to induce a measurable but not complete reduction in MTS metabolic activity. Both staurosporine and CoCl_2_ treatments (Figure 1A,B) reduced MTS metabolic activity in HUVECs, whereas PEG35 significantly attenuated this reduction under all tested stress scenarios. PEG35 alone did not alter basal MTS metabolic activity.

**Figure 1:**
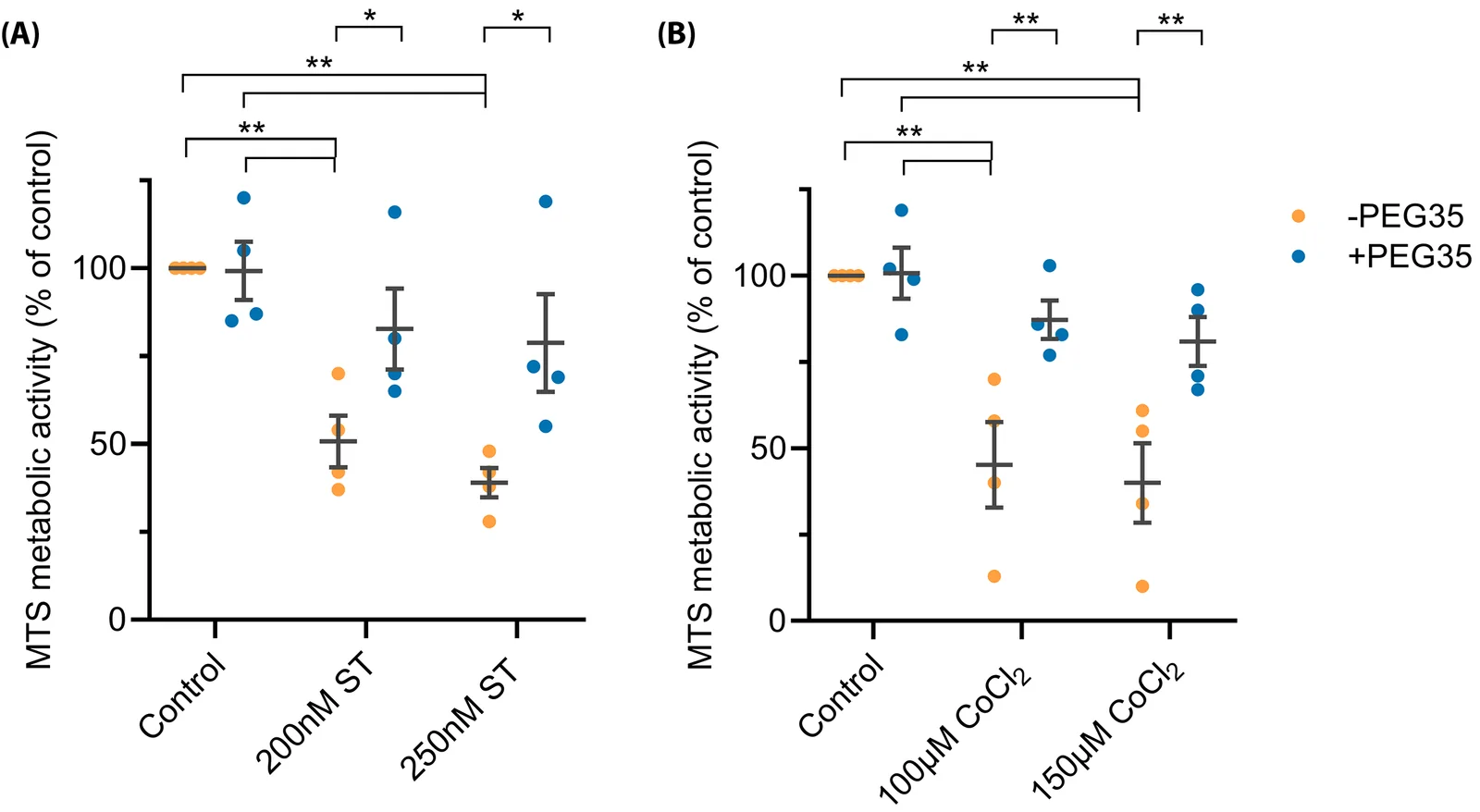
PEG35 preserves endothelial cell metabolic activity under apoptotic and hypoxia-mimetic stress HUVECs were pre-treated with 4% PEG35 for 30 min and then exposed for 4 h to (**A**) 200 or 250 nM staurosporine or (**B**) 100 or 150 μM CoCl_2_. MTS metabolic activity was expressed as a percentage of untreated control cells. Each dot represents one independent experiment calculated as the mean of technical triplicates. Data are shown as mean ± SEM from four independent experiments. *P<0.05, **P<0.01. Asterisks indicate the comparisons shown by brackets. PEG35, 35-kDa polyethylene glycol; ST, staurosporine; CoCl_2_, cobalt chloride.

### PEG35 counteracted endothelial cell barrier dysfunction induced by inflammatory and eGCX-disrupting stimuli

Having demonstrated that PEG35 confers significant protection against apoptosis and hypoxia-induced injury in endothelial cells, we next aimed to study the potential barrier-stabilizing role of PEG35. For that, we challenged HUVEC monolayers with inflammatory and eGCX-disrupting stimuli and evaluated barrier integrity through FITC-dextran permeability and TEER measurements (Figure 2). Exposure to thrombin, TNFα, and IFNγ significantly increased vascular permeability, as evidenced by elevated FITC-dextran concentrations across the HUVEC monolayer compared with untreated controls (Figure 2A). PEG35 treatment prior to injurious stimulation significantly attenuated the increase in permeability induced by these stimuli, with values approaching those of untreated control cells.

**Figure 2:**
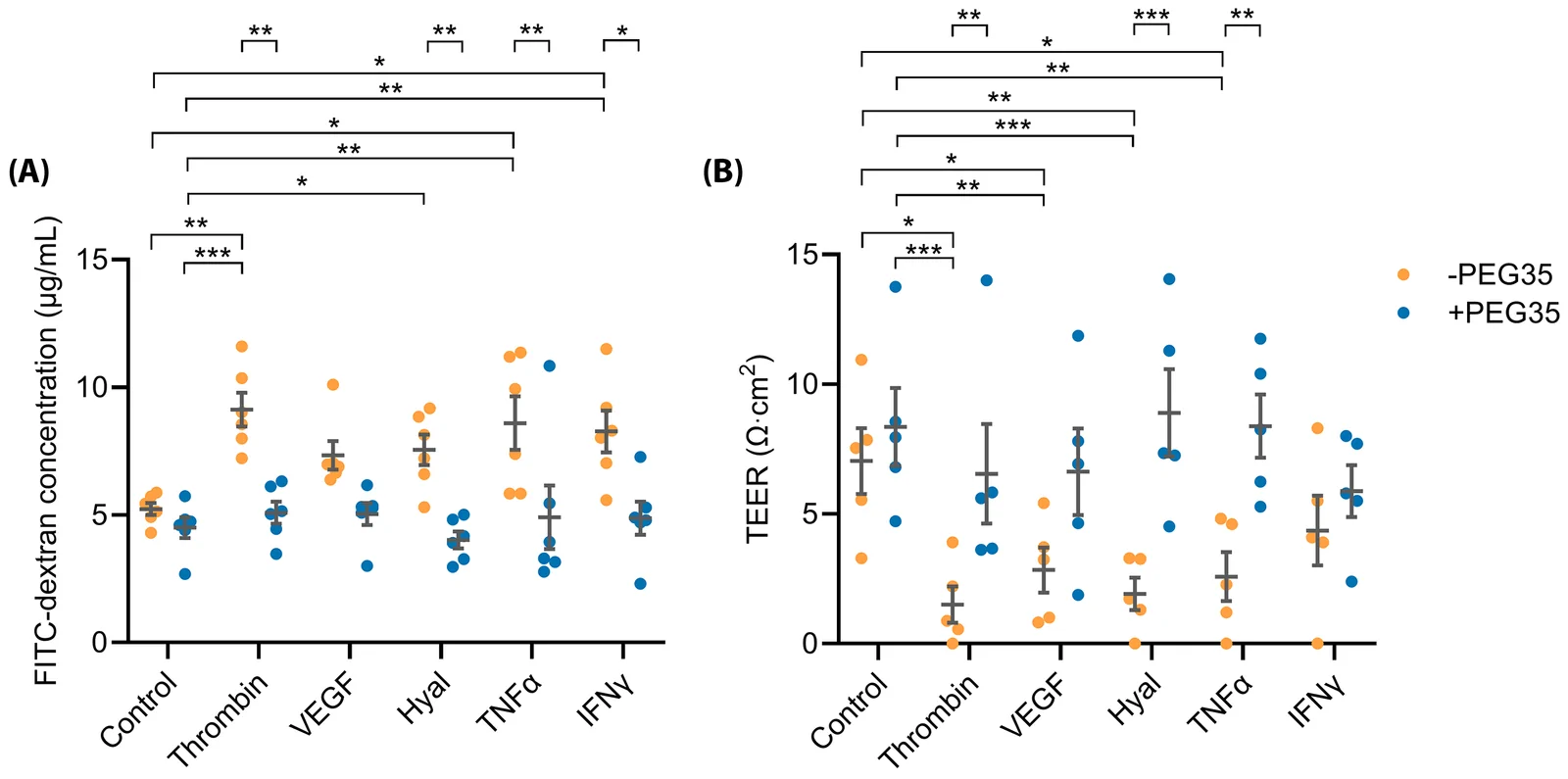
PEG35 attenuated endothelial barrier dysfunction induced by selected inflammatory and eGCX-disrupting stimuli (**A**) Endothelial permeability was assessed by measuring FITC-dextran translocation across HUVEC monolayers treated for 2 h with 2 U/ml thrombin, 50 ng/ml VEGF, 15 U/ml hyaluronidase, 60 ng/ml TNFα, and 20 ng/ml IFNγ. In selected experimental groups, HUVECs were treated with 4% PEG35 for 1 h before stimulation. (**B**) TEER was measured under the same experimental conditions. Each dot represents one independent experiment, with technical duplicates averaged before analysis. Data are shown as mean ± SEM from six independent experiments for the permeability assay and five independent experiments for the TEER assay. *P<0.05, **P<0.01, ***P<0.001. Asterisks indicate the comparisons shown by brackets. PEG35, 35-kDa polyethylene glycol; VEGF, vascular endothelial growth factor; Hyal, hyaluronidase; TNFα, tumor necrosis factor α; IFNγ, interferon γ; TEER, *trans*-endothelial electrical resistance.

TEER analysis consistently revealed a significant reduction in endothelial resistance following treatment with all agents except IFNγ, which showed a non-significant downward trend (Figure 2B). PEG35 treatment prior to injurious stimulation preserved TEER values in cells stimulated with thrombin, hyaluronidase, and TNFα, attenuating the stimulus-induced loss of endothelial resistance. However, in response to VEGF, PEG35 elicited only a partial, non-significant recovery in TEER. PEG35 alone did not alter endothelial permeability or TEER. Collectively these findings suggest that PEG35 can counteract endothelial barrier dysfunction induced by inflammatory and eGCX-degrading factors.

### PEG35 mitigated endothelial cell injury associated with postreperfusion plasma from pancreas transplant recipients

To evaluate the translational relevance of our findings, we investigated whether PEG35 could limit endothelial cells from injury during exposure to plasma derived from pancreas transplant patients collected 10 min after pancreas reperfusion (PR plasma), a critical timepoint known to reflect peak IRI (Figure 3).

**Figure 3:**
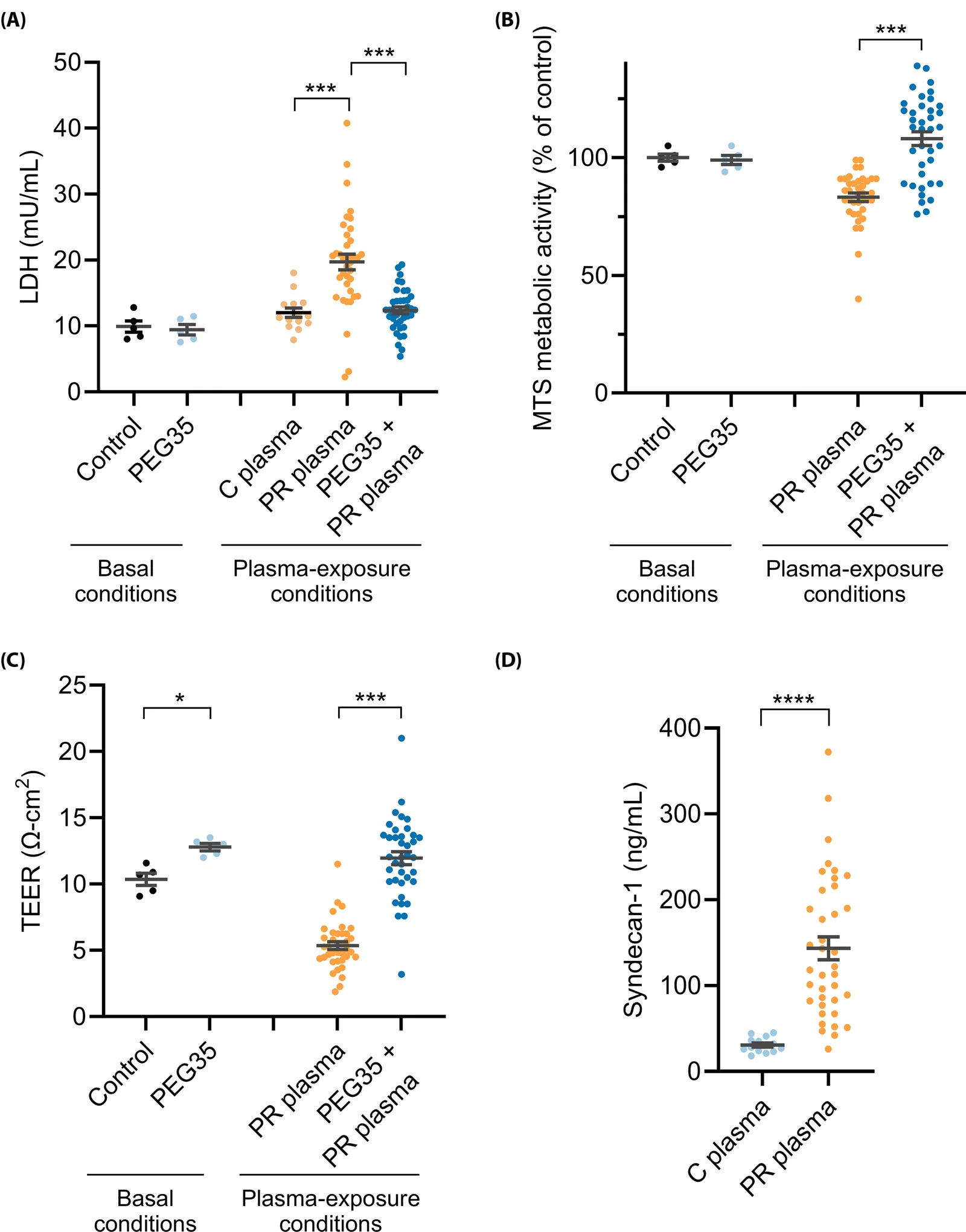
PEG35 mitigated endothelial cell injury associated with postreperfusion plasma from pancreas transplant recipients HUVECs were treated with 5% human PR plasma collected 10 min after reperfusion of the pancreas. Where indicated, cells were exposed to 4% PEG35 for 30 min prior to stimulation with PR plasma. (**A**) LDH activity in culture supernatants following 24-h exposure to healthy-control plasma or PR plasma in the absence or presence of PEG35. LDH activity is expressed in mU/ml. (**B**) MTS metabolic activity following 24-h exposure to PR plasma in the absence or presence of PEG35, expressed as a percentage of the untreated control. (**C**) TEER values in HUVEC monolayers following 2-h exposure to PR plasma in the absence or presence of PEG35, expressed as Ω·cm^2^. (**D**) Plasma syndecan-1 concentrations in healthy controls and pancreas transplant recipients 10 min after graft reperfusion, expressed in ng/ml. Each dot represents one experimental run for untreated control and PEG35-alone conditions or one individual plasma sample for plasma-exposure conditions. Technical duplicates were averaged before analysis. Data are shown as mean ± SEM. Asterisks indicate the significant prespecified comparisons shown by brackets. *P<0.05, ***P<0.001, ****P<0.0001. Asterisks indicate the comparisons shown by brackets. PEG35, 35-kDa polyethylene glycol; PR plasma, postreperfusion plasma; LDH, lactate dehydrogenase; TEER, *trans*-endothelial electrical resistance; C plasma, control plasma.

PR plasma induced significantly greater LDH release than plasma from healthy controls (Figure 3A), indicating increased endothelial cell membrane damage under post-reperfusion plasma exposure. Pretreatment with PEG35 significantly reduced LDH release in cells exposed to the same individual PR plasma samples. In contrast, PEG35 alone did not significantly alter basal LDH release. These findings support a protective effect of PEG35 against PR plasma-associated endothelial cell injury.

Exposure to PR plasma was also associated with reduced MTS metabolic activity. Pretreatment with PEG35 significantly attenuated this reduction when the same individual PR plasma samples were assessed in the absence or presence of PEG35 (Figure 3B). PEG35 alone did not significantly alter basal MTS metabolic activity, suggesting that PEG35 helps maintain endothelial cell metabolic activity during PR plasma exposure without producing a detectable effect under basal conditions.

TEER analysis showed that endothelial cells pre-treated with PEG35 maintained significantly higher electrical resistance during exposure to the same individual PR plasma samples than cells exposed to PR plasma alone (Figure 3C). PEG35 alone also significantly increased basal TEER compared with untreated cells, indicating a barrier-reinforcing effect under basal conditions. Therefore, the higher TEER values observed in the PEG35 + PR plasma condition may reflect both PEG35-induced enhancement of basal endothelial resistance and attenuation of PR plasma-associated barrier disruption.

To further explore the mechanisms underlying PEG35-mediated endothelial protection in this clinical IRI context, we examined the levels of syndecan-1, a key component of the eGCX, in PR plasma samples. Our analysis revealed a significant increase in syndecan-1 plasma levels in pancreas transplant patients compared with healthy controls, indicating substantial eGCX disruption induced by early graft reperfusion (Figure 3D). This observation reinforces the hypothesis that the circulating factors present at this early reperfusion stage may contribute to endothelial dysfunction, at least in part, through eGCX shedding.

### PEG35 reduced NFκB p65 nuclear translocation in stimulated endothelial cells

An anti-inflammatory role for PEG35 has previously been described in pancreatic epithelial cells in response to pro-inflammatory stimuli [37]. To investigate the molecular mechanism underlying the protective effect of PEG35 in the context of endothelial injury, we performed immunofluorescence staining to assess the activation of the NF-κB pathway in HUVECs in response to pro-inflammatory and barrier-disrupting agents.

As shown in Figure 4A, stimulation with thrombin, hyaluronidase, and TNFα promoted nuclear localization of the p65 subunit of NF-κB compared with control cells, with TNFα inducing the most pronounced response. Consistent with the representative images, quantitative analysis of the percentage of cells showing nuclear p65 localization confirmed a significant increase after stimulation (Figure 4B). PEG35 pretreatment significantly reduced stimulus-induced p65 nuclear translocation in response to thrombin, hyaluronidase, and TNFα. These findings support an attenuation of NF-κB-related inflammatory activation by PEG35 under these injurious conditions.

**Figure 4:**
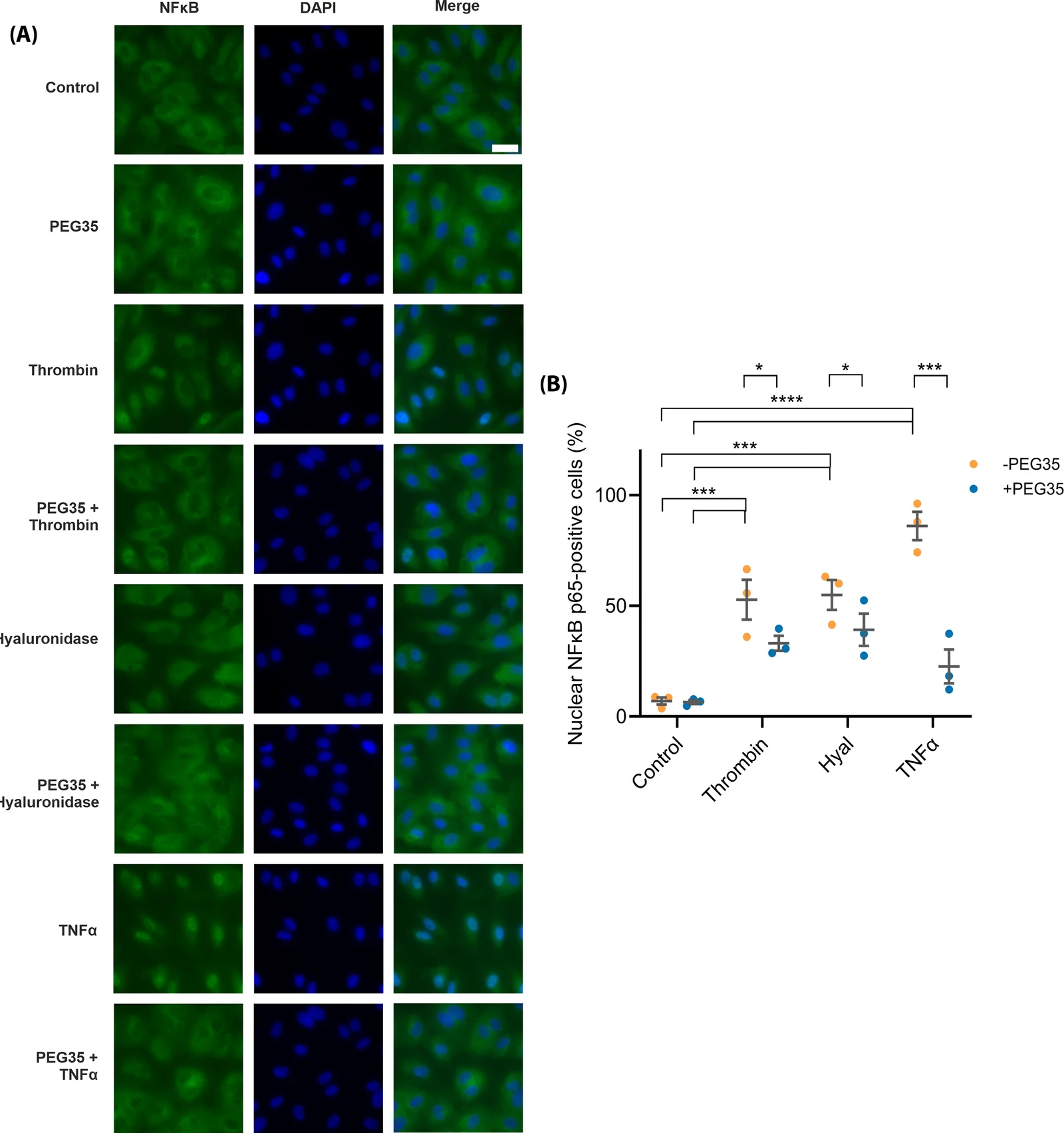
PEG35 attenuated the nuclear translocation of the p65 subunit of NF-κB pathway in endothelial cells stimulated with thrombin, TNFα, and hyaluronidase (**A**) Representative images of immunofluorescence staining showing the subcellular localization of the NF-κB p65 subunit (green) and nucleus stained with DAPI (blue) in endothelial cells under different stimuli. Scale bar, 10 μm. (**B**) Quantification of NF-κB p65 nuclear translocation, expressed as the percentage of cells showing nuclear p65 localization. Each dot represents one independent experiment, calculated as the mean of five randomly selected fields per condition. Data are expressed as mean ± SEM from three independent experiments. *P<0.05, ***P<0.001, ****P<0.0001. Asterisks indicate the comparisons shown by brackets. PEG35, 35-kDa polyethylene glycol; TNFα, tumor necrosis factor α; NF-κB, nuclear factor kappa B.

### PEG35 modulated inflammatory and endothelial activation-related gene expression under selected injurious conditions

To further elucidate the mechanisms underlying PEG35-mediated protection of the endothelial barrier, we assessed the expression of inflammatory and vascular function related genes in HUVECs stimulated with thrombin, hyaluronidase, or TNFα (Figure 5). The transcriptional responses differed according to the stimulus and gene analyzed.

**Figure 5:**
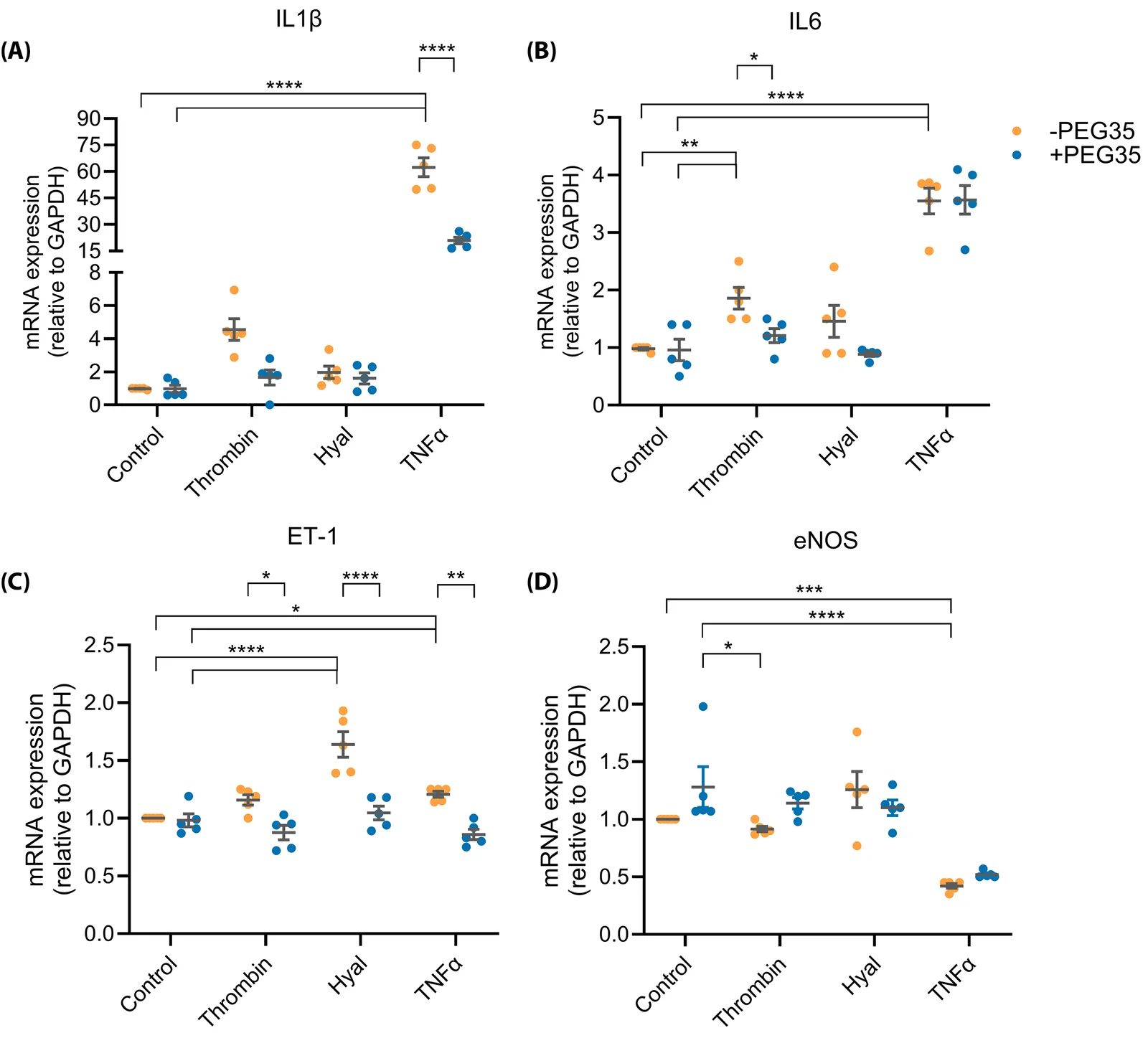
PEG35 exerts stimulus-dependent effects on inflammatory and endothelial gene expression Effects of PEG35 on (**A**) IL1β, (**B**) IL6, (**C**) ET-1, and (**D**) eNOS gene expression in HUVECs stimulated with thrombin, TNFα, and hyaluronidase for 3 h. mRNA levels of these genes were normalized to GAPDH. Expression values were calculated using the ΔCt method and presented as fold change relative to untreated control cells. Each dot represents one independent experiment, with technical duplicates averaged before analysis. Data are expressed as mean ± SEM from five independent experiments and shown as fold change relative to untreated control cells. *P<0.05, **P<0.01, ***P<0.001, ****P<0.0001. Asterisks indicate the comparisons shown by brackets. IL1β, interleukin 1β, IL6, interleukin 6; ET-1, endothelin-1; eNOS, endothelial nitric oxide synthase; PEG35, 35-kDa polyethylene glycol; TNFα, tumor necrosis factor α; Hyal, hyaluronidase.

TNFα markedly increased IL1β expression compared with untreated control cells, and PEG35 treatment significantly attenuated this induction (Figure 5A). No significant PEG35-associated differences were observed in IL1β expression under thrombin or hyaluronidase stimulation.

IL6 expression was significantly increased by thrombin and TNFα compared with untreated control cells (Figure 5B). PEG35 pretreatment significantly attenuated the thrombin-induced increase in IL6 but did not significantly modify the response to TNFα. Hyaluronidase did not alter IL6 expression under the conditions tested.

ET-1 expression was significantly increased by hyaluronidase and TNFα compared with untreated control cells, whereas thrombin did not significantly modify ET-1 expression relative to control (Figure 5C). Nevertheless, PEG35 pretreatment significantly reduced ET-1 expression relative to each corresponding stimulus-alone condition, including thrombin, hyaluronidase, and TNFα. Thus, the effect of PEG35 on ET-1 expression was stimulus-dependent and, in the thrombin condition, should be interpreted as modulation relative to thrombin alone, rather than attenuation of a significant thrombin-induced increase.

TNFα significantly reduced eNOS expression compared with untreated control cells, whereas thrombin and hyaluronidase had no significant effect under the experimental conditions tested (Figure 5D). PEG35 pretreatment did not significantly modify eNOS expression in response to any of the stimuli.

### PEG35 modulated intercellular junction gene expression in stimulated endothelial cells

In a separate set of experiments, we explored whether PEG35 also contributes to the maintenance of structural components involved in endothelial barrier function. The endothelial barrier relies on both tight and adherens junctions to sustain cohesive cell-cell interactions and ensure vascular integrity. As shown in Figure 6, the transcriptional responses varied according to the stimulus and junctional component analyzed.

**Figure 6:**
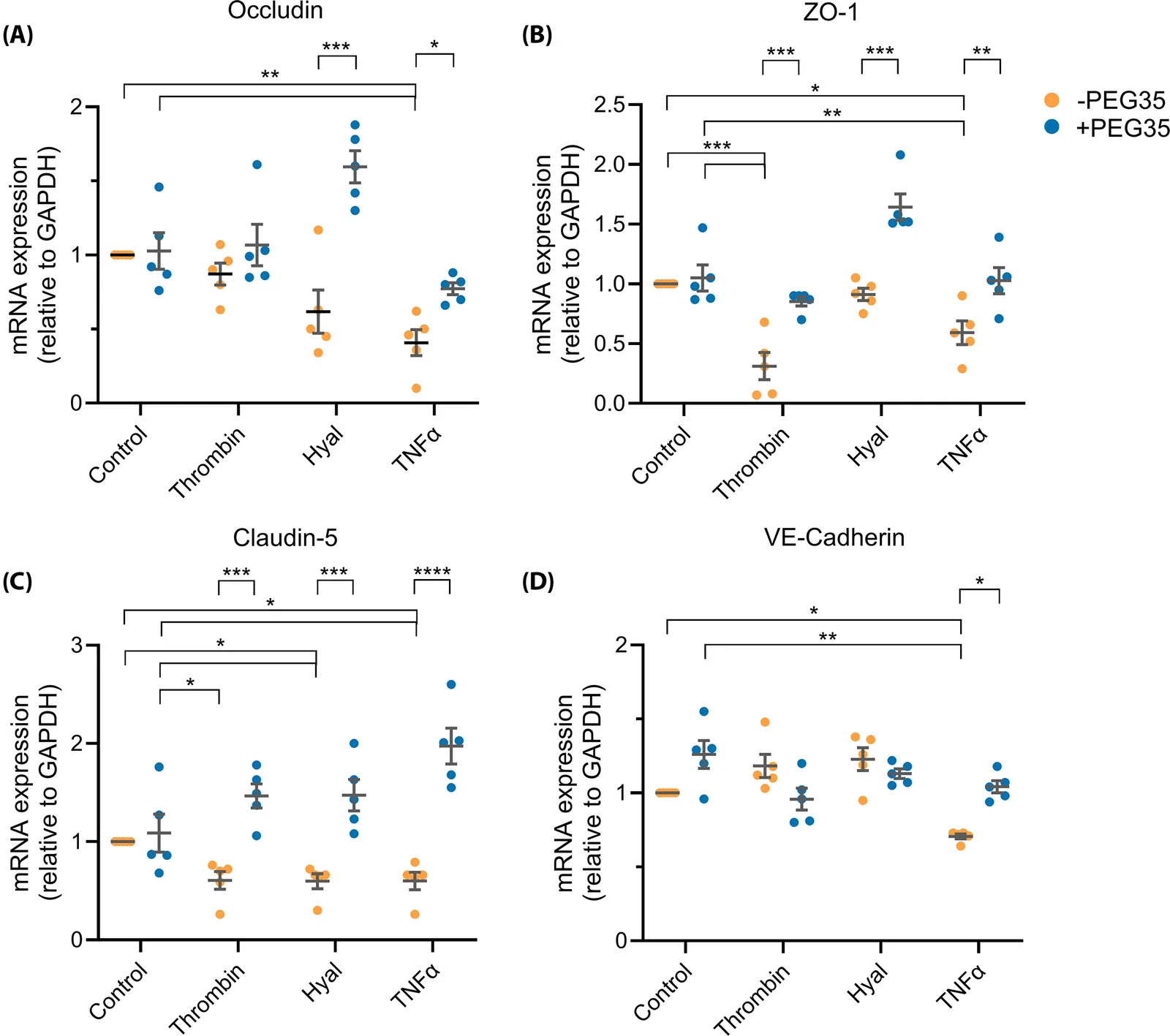
PEG35 limited injury-induced down-regulation of intercellular junction genes (**A**) Occludin, (**B**) ZO-1, (**C**) Claudin-5, and (**D**) VE-cadherin in HUVECs stimulated with thrombin, TNFα, and hyaluronidase for 3 h. mRNA levels were normalized to GAPDH. Expression values were calculated using the ΔCt method and presented as fold change relative to untreated control cells. Each dot represents one independent experiment, with technical duplicates averaged before analysis. Data are expressed as mean ± SEM from five independent experiments and shown as fold change relative to untreated control cells. *P<0.05, **P<0.01, ***P<0.001, ****P<0.0001. Asterisks indicate the comparisons shown by brackets. ZO-1, zona occludens 1; VE-cadherin, vascular endothelial cadherin; PEG35, 35-kDa polyethylene glycol; TNFα, tumor necrosis factor α; Hyal, hyaluronidase.

TNFα significantly reduced occludin expression compared with untreated control cells, and PEG35 pretreatment significantly attenuated this reduction (Figure 6A). Occludin levels were also higher in PEG35-pretreated cells exposed to hyaluronidase than in cells treated with hyaluronidase alone, although hyaluronidase did not significantly modify occludin expression relative to control. Neither thrombin nor PEG35 pretreatment significantly affected occludin expression under thrombin stimulation.

ZO-1 expression was significantly reduced by thrombin and TNFα compared with untreated control cells (Figure 6B). PEG35 pretreatment increased ZO-1 expression relative to each corresponding stimulus-alone condition. Because hyaluronidase did not significantly alter ZO-1 expression compared with control cells, the higher expression observed in PEG35-pretreated cells exposed to hyaluronidase indicates modulation by PEG35 rather than attenuation of an established hyaluronidase-induced reduction.

Claudin-5 expression was significantly reduced by hyaluronidase and TNFα, and PEG35 pretreatment significantly attenuated both reductions (Figure 6C). Although thrombin did not significantly alter claudin-5 expression relative to control, expression was higher in PEG35 pretreated cells exposed to thrombin than in cells treated with thrombin alone.

VE-cadherin expression was significantly reduced by TNFα, whereas thrombin and hyaluronidase produced no significant changes relative to control cells (Figure 6D). PEG35 pretreatment attenuated the TNFα-associated reduction but did not modify VE-cadherin expression under thrombin or hyaluronidase stimulation.

Overall, these findings indicate that PEG35 modulated intercellular junction gene expression in a stimulus- and target-dependent manner. Differences between stimulus-alone and PEG35-pretreated conditions were interpreted as attenuation of junctional loss only when the corresponding stimulus produced a significant reduction relative to untreated control cells.

### PEG35 helped maintain intercellular junction protein expression under selected injurious conditions

To determine whether the transcriptional effects on junctional components were also reflected at the protein level, we evaluated occludin and claudin-5 expression by Western blot (Figure 7). Protein levels of both occludin and claudin-5 were significantly reduced following stimulation with thrombin, hyaluronidase, and TNFα. PEG35 treatment prior to stimulation significantly attenuated the reduction of both proteins' expression in response to hyaluronidase and TNFα. However, PEG35 did not significantly prevent the thrombin-induced reduction in either protein under the experimental conditions tested. These findings indicate that PEG35 helped preserve selected intercellular junction proteins in a stimulus-dependent manner, particularly under eGCX-disrupting and inflammatory conditions.

**Figure 7:**
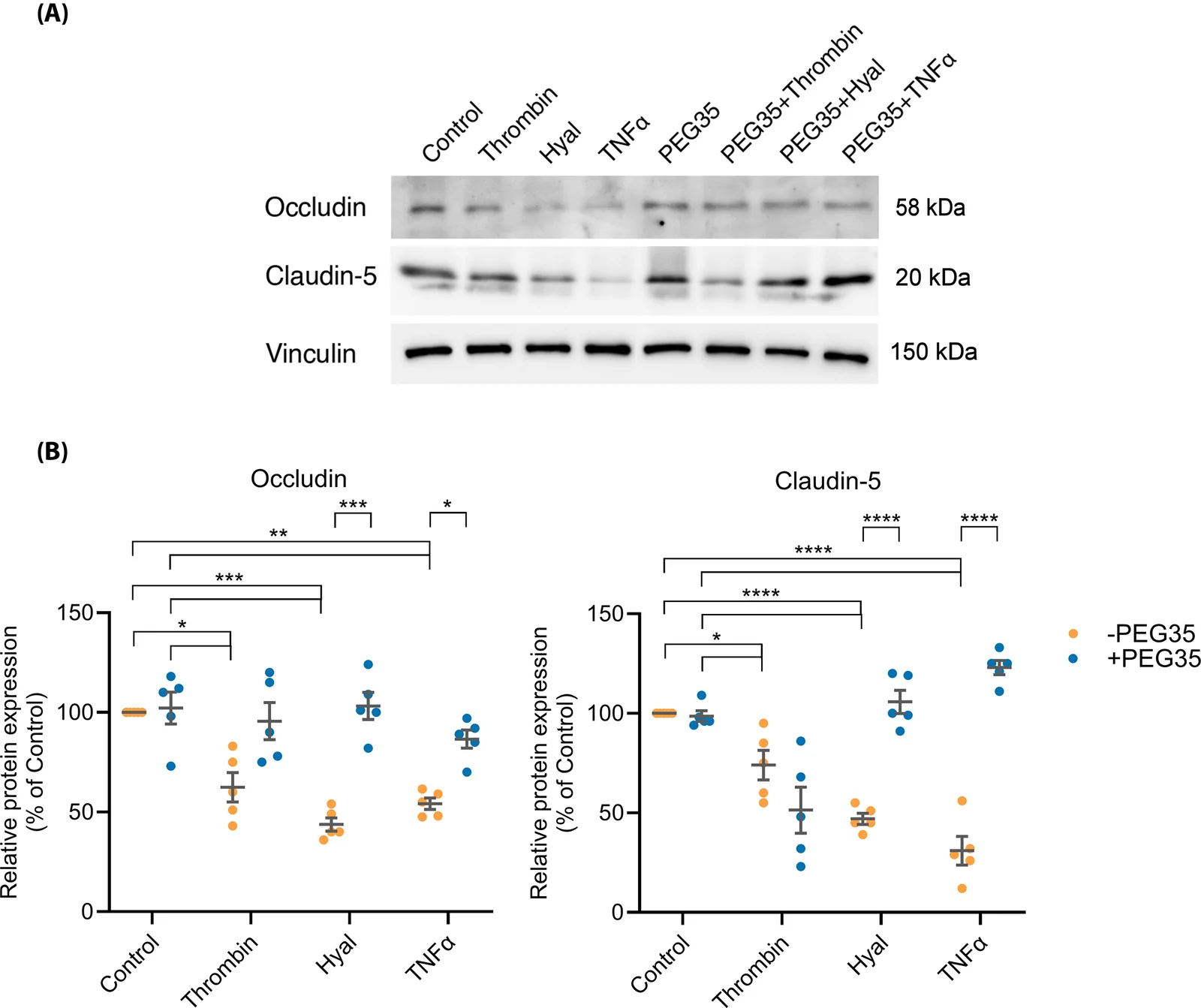
PEG35 preserved the expression of intercellular junction proteins in endothelial cells stimulated with thrombin, TNFα, and hyaluronidase Western blot analysis was performed on lysates from HUVECs treated for 4 h with 2 U/ml thrombin, 60 ng/ml TNFα, or 15 U/ml hyaluronidase. In selected experimental groups, HUVECs were treated with 4% PEG35 for 30 min prior to stimulation. (**A**) The expression levels of the tight junction proteins occludin and claudin-5 were assessed by Western blot. Representative immunoblots are shown. (**B**) Densitometric quantification of occludin and claudin-5 expression was performed by using ImageJ software. Protein levels were normalized to vinculin and expressed as a percentage relative to the untreated control group. Each dot represents one independent experiment. Data are presented as mean ± SEM from five independent experiments. *P<0.05, **P<0.01, ***P<0.001, ****P<0.0001. Asterisks indicate the comparisons shown by brackets. PEG35, 35-kDa polyethylene glycol; TNFα, tumor necrosis factor α; Hyal, hyaluronidase.

### PEG35 limited the loss of key eGCX components in endothelial cells exposed to eGCX- disrupting enzymes

We then studied the effect of PEG35 on the preservation of eGCX following enzymatic degradation (Figure 8). Specifically, we assessed the expression of two major eGCX components, syndecan-1 (Figure 8A) and HS (Figure 8B), using immunofluorescence staining in HUVECs treated with MMP7 or heparinase III, respectively. We observed a marked decrease in the percentage of stained area for both HS and syndecan-1 following enzyme treatment. This loss was significantly attenuated in cells previously treated with PEG35, suggesting that PEG35 helps preserve eGCX-associated components under enzymatic degradation conditions. These findings support the role of PEG35 in maintaining endothelial surface structures, critical for vascular homeostasis and barrier function.

**Figure 8:**
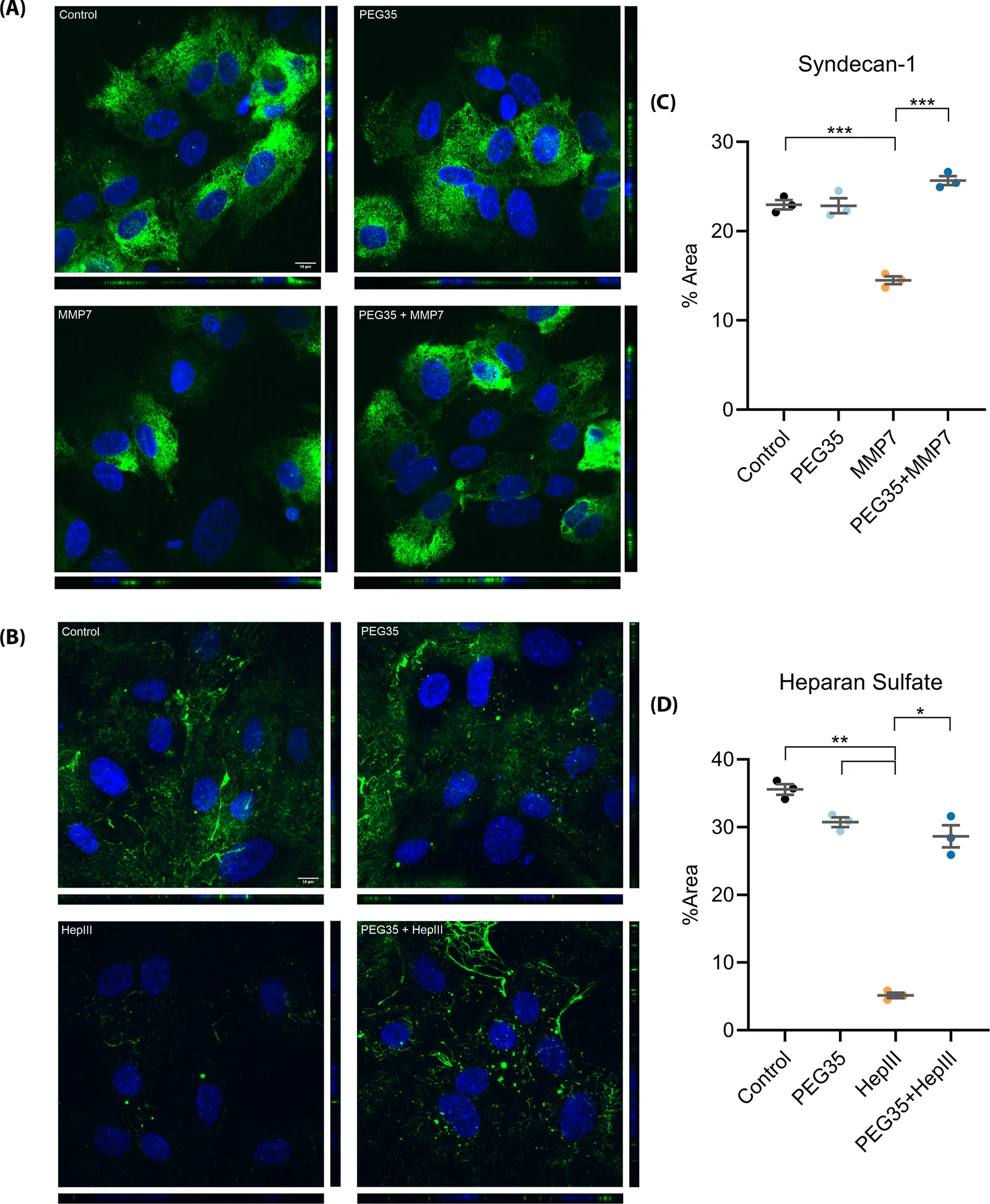
PEG35 limited the loss of eGCX components in endothelial cells exposed to eGCX-degrading enzymes Representative maximum-intensity projections of confocal z-stacks showing (**A**) syndecan-1 and (**B**) HS staining in HUVECs under basal conditions, after treatment with PEG35, after exposure to eGCX-disruptive enzymes, and after PEG35 treatment followed by enzymatic stimulation. Target eGCX components are shown in green, and nuclei are stained with DAPI (blue). Quantification of the stained area for each eGCX component is shown in the scatter dot plots (**C,D**). Each dot represents one independent experiment, calculated as the mean of analyzed fields and technical replicates for each condition. Data are shown as mean ± SEM from three independent experiments. Scale bars: 10 μm. *P<0.05, **P<0.01, ***P<0.001. Asterisks indicate the comparisons shown by brackets. PEG35, 35-kDa polyethylene glycol; MMP7, matrix metalloproteinase 7; Hep III, heparinase III.

## Discussion

Human endothelial cells are multifunctional components of the vascular system, forming a barrier that lines blood vessels and actively secretes a wide range of bioactive mediators to preserve vascular homeostasis and inhibit thrombotic events [34]. Under pathological conditions, including infections, oxidative stress, or IRI, the endothelium can transition to a dysfunctional state characterized by eGCX degradation, reduced nitric oxide bioavailability, and disruption of adhesive and junctional integrity, thereby enhancing endothelial barrier dysfunction [40]. In parallel, the activation of adhesion molecules and dysregulated cytokine and chemokine expression contribute to the progression of vascular dysfunction.

The present study shows that PEG35 helps preserve endothelial integrity under several injurious conditions. PEG35 attenuated reductions in MTS metabolic activity under apoptotic and hypoxia-mimetic stress, reduced FITC-dextran permeability, and maintained TEER in endothelial monolayers exposed to inflammatory, thrombotic, and eGCX-disrupting stimuli. These functional effects were accompanied by modulation of key eGCX and junctional components and attenuation of NF-κB-related inflammatory activation. Together, these findings support a multifactorial model of PEG35-mediated endothelial protection involving preservation of the endothelial surface layer, maintenance of paracellular junctional integrity, and regulation of inflammatory signalling.

These observations are consistent with previous studies showing barrier-protective effects of high-molecular-weight PEGs. For example, PEG15–20 preserved the barrier function of human lung endothelial cells exposed to viral factors and pro-inflammatory stimuli, an effect linked to stabilization of the actin cytoskeleton, preservation of cortical actin structures, and inhibition of stress fiber formation [19]. A complementary biophysical mechanism may also contribute, since high-molecular-weight PEGs, including PEG35, have been shown to interact with membrane glycerophospholipids and modulate lipid monolayer stability under defined experimental conditions [41].

In our study, PEG35 also showed protective effects against circulating factors derived from a clinically relevant IRI context. The use of plasma collected from pancreas transplant recipients 10 min after reperfusion provides a translationally relevant model of the complex pathophysiological milieu associated with early reperfusion injury. This environment, characterized by increased vascular permeability, elevated levels of pro-inflammatory cytokines, reactive oxygen species, and other damaging mediators, promotes endothelial dysfunction and compromises barrier integrity [42]. In this context, PEG35 pretreatment reduced LDH release and helped maintain MTS metabolic activity in endothelial cells exposed to PR plasma samples. Moreover, PR plasma induced greater LDH release than plasma from healthy controls, supporting the presence of circulating factors associated with endothelial cell injury during early reperfusion. Together, these findings support a protective effect of PEG35 against PR plasma-associated endothelial injury.

The TEER results from the plasma-exposure experiments should, however, be interpreted with caution. Endothelial cells pre-treated with PEG35 maintained higher TEER values during exposure to PR plasma samples than cells exposed to PR plasma alone. However, PEG35 alone increased basal TEER without significantly affecting LDH release or MTS metabolic activity, suggesting that the polymer may reinforce endothelial electrical resistance under basal conditions. Therefore, the higher TEER values observed in cells pre-treated with PEG35 and subsequently exposed to PR plasma may reflect both this basal barrier-reinforcing effect and attenuation of PR plasma-associated barrier disruption. The present experimental design does not allow these two components to be fully dissociated. Furthermore, despite the translational relevance of the PR plasma model, this *in vitro* system does not reproduce the hemodynamic, cellular, and immunological complexity of the *in vivo* reperfusion environment.

At the molecular level, PEG35 limited NF-κB activation, a central regulator of endothelial inflammatory responses. Specifically, PEG35 reduced nuclear translocation of the p65 subunit in HUVECs stimulated with thrombin, hyaluronidase, and TNFα. PEG35 also attenuated selected stimulus-induced changes in IL1β, IL6, and ET-1 expression, although the pattern differed according to the stimulus and gene analyzed. Together, these findings support a stimulus-dependent anti-inflammatory action of PEG35 rather than a uniform effect across all inflammatory markers and experimental conditions. This interpretation is consistent with previous evidence from our group showing that intravenous administration of PEG35 reduced inflammatory damage in an experimental model of acute necrotizing pancreatitis, even when administered after the onset of systemic manifestations [43]. In contrast, PEG35 did not significantly modify eNOS expression under the experimental conditions tested. TNFα significantly reduced eNOS expression, but PEG35 pretreatment did not attenuate this response, whereas thrombin and hyaluronidase did not significantly alter eNOS expression. Given that eNOS contributes to nitric oxide production, vascular tone, anti-inflammatory signaling [44], and endothelial barrier integrity [45], these findings indicate that the endothelial-protective effects of PEG35 observed in the present study were not associated with detectable preservation of eNOS expression.

Endothelial barrier function and vascular permeability are regulated by intercellular junctions that control the passage of plasma components [46]. In the present study, PEG35 modulated the expression of tight- and adherens-junction components in a stimulus- and target-dependent manner. Its transcriptional effects were most consistently observed during TNFα-induced injury, although significant effects on selected junctional components were also detected under thrombin and hyaluronidase stimulation. Importantly, some differences between stimulus-alone and PEG35-pretreated conditions occurred even when the stimulus did not significantly reduce expression relative to untreated control cells. These findings should therefore be interpreted as stimulus-dependent modulation by PEG35 rather than uniform preservation of injury-induced junctional loss. At the protein level, PEG35 attenuated the TNFα- and hyaluronidase-associated reductions in occludin and claudin-5, providing complementary evidence of protection of selected junctional components. PEG35 did not significantly modify the thrombin-induced reduction in either protein, further indicating that its influence on junctional protein expression depends on the injurious stimulus. This junctional modulation may be particularly relevant in the context of eGCX disruption, given the functional relationship between the eGCX and intercellular junctions [47]. Tight junction proteins, including occludin, claudin-5, and ZO-1, contribute to the regulation of paracellular permeability [48], whereas VE-cadherin adherens junctions provide mechanical stability through their association with the actin cytoskeleton [49].

The mechanisms underlying these stimulus and target-dependent effects remain to be established. They may involve attenuation of injury-related inflammatory signaling, including NF-κB activation, together with biophysical effects at the endothelial surface or membrane interface and cytoskeletal stabilization, as previously described for other high-molecular-weight PEGs [19,41]. Preservation of eGCX may also indirectly support junctional stability by maintaining the structural and signaling environment of the endothelial surface. However, because junctional protein localization, cytoskeletal organization, and junction regulatory signaling were not examined, these possibilities should be considered mechanistic hypotheses rather than demonstrated direct effects of PEG35.

PEG35 also limited the loss of critical components of the eGCX, such as syndecan-1 and HS, following enzymatic degradation by MMP7 and heparinase III, respectively. The reduced loss of immunostained area suggests that PEG35 helps preserve the endothelial surface layer under eGCX-disrupting conditions. The precise mechanism underlying this effect remains to be determined, but it may involve biophysical protection of the endothelial surface, attenuation of injury-related signaling, or both. Importantly, the present study does not establish whether PEG35 directly inhibits MMP7 or heparinase III activity, binds to specific eGCX components, or forms a protective pericellular layer. This finding is potentially relevant, as eGCX degradation has been linked to vascular leakage, edema, inflammation, and endothelial dysfunction. The increased syndecan-1 concentrations detected in PR plasma further support the relevance of eGCX shedding in the clinical reperfusion context evaluated in the present study, although they do not establish that syndecan-1 or other eGCX-derived products were directly responsible for the endothelial effects of PR plasma.

The cellular localization of PEG35 in the present HUVEC model remains unresolved. The current data are compatible with effects on the endothelial surface layer, since PEG35 maintained barrier properties and limited the reduction of eGCX components. However, intracellular or membrane-associated actions cannot be excluded. Previous studies indicate that PEG-cell interactions depend on molecular weight and experimental context. Cellular uptake of PEGs has been reported to occur by passive diffusion for lower-molecular-weight PEGs and through a combination of passive diffusion and endocytic mechanisms for higher-molecular-weight PEGs under specific experimental conditions [50]. In addition, 2-kDa PEG has been detected in cytoplasmic and nuclear compartments and associated with adaptive metabolic or autophagic responses [51]. These findings cannot be directly extrapolated to PEG35 or HUVECs, but they support the concept that PEGs may interact dynamically with cells rather than acting exclusively as extracellular inert polymers. In the present study, direct PEG35 localization in HUVECs was not assessed. Therefore, the present data do not allow us to determine whether PEG35 remains extracellular, associates with the endothelial surface or eGCX, or is internalized. In line with this concept, previous work by our group showed that PEG35 enhanced exosome uptake in macrophages and epithelial cells while attenuating acute pancreatitis-derived exosome- or LPS-induced inflammatory activation, including IL1β expression and NF-κB p65 nuclear translocation [38]. These findings suggest that PEG35 can influence cell-associated uptake processes and downstream inflammatory signaling. In the present endothelial model, PEG35-mediated protection may therefore arise from a combination of effects on the endothelial surface layer, intercellular junctions, and inflammatory signaling pathways. Nevertheless, this possibility requires direct evaluation. Future studies using labeled PEG35, quantitative imaging, and subcellular fractionation will be required to define its cellular localization and determine how this relates to the observed endothelial protective effects.

The endothelial phenotype of the HUVEC cultures was supported by cobblestone-like morphology and CD31 staining. However, additional endothelial markers or functional assays would provide a more comprehensive characterization of the cell model. Moreover, although the present data support an endothelial-protective effect of PEG35, the biological behavior of PEGs depends on molecular weight, formulation, route of administration, dose, and exposure duration. A complete PEG35 concentration-response analysis was not performed across all endothelial readouts; therefore, the present study does not define the minimal effective concentration or the full concentration-dependent profile of PEG35 in HUVECs. Furthermore, this single-exposure *in vitro* study should not be interpreted as evidence of long-term systemic safety. Future studies should assess PEG35 localization, biodistribution, potential accumulation, concentration-dependent effects, and safety under repeated or systemic exposure conditions.

Taken together, these findings support the potential of PEG35 to preserve endothelial barrier integrity under selected injurious conditions through effects involving the endothelial surface layer, intercellular junctions, and inflammatory signaling.

## Supplementary Material

Supplementary Figure S1

## Data Availability

All data included in the present study are available upon request by contact with the corresponding author.

## Open Access

Open access for this article was enabled by the participation of Consejo Superior de Investigaciones Cientificas (CSIC) in an all-inclusive Read & Publish agreement with Portland Press and the Biochemical Society.

## Abbreviations

Ct: threshold cycle
CoCl_2_: cobalt chloride
eGCX: endothelial glycocalyx
EGM-2: endothelial cell growth medium-2
ELISA: enzyme-linked immunosorbent assay
FBS: fetal bovine serum
FITC: fluorescein isothiocyanate
GAGs: glycosaminoglycans
HS: heparan sulfate
HUVECs: human umbilical vein endothelial cells
IRI: ischemia-reperfusion injury
LDH: lactate dehydrogenase
MMP7: matrix metalloproteinase 7
NFκB: nuclear factor kappa B
PEG: polyethylene glycol
PEG35: polyethylene glycol 35
TEER: *trans*-endothelial electrical resistance
TNFα: tumor necrosis factor α
VE-cadherin: vascular endothelial cadherin
ZO-1: zona occludens-1
