# Supplementary material for: Polyethylene glycol 35 preserves endothelial barrier integrity by stabilizing the glycocalyx and intercellular junctions
Source: Biosci Rep. 2026 Nov 11;46(11):BSR20260686. doi: 10.1042/BSR20260686 (PMC13768145; doi:10.1042/BSR20260686)
Supplement: Supplementary file 1 [file BSR-2026-0686_supp.pdf]

a

b

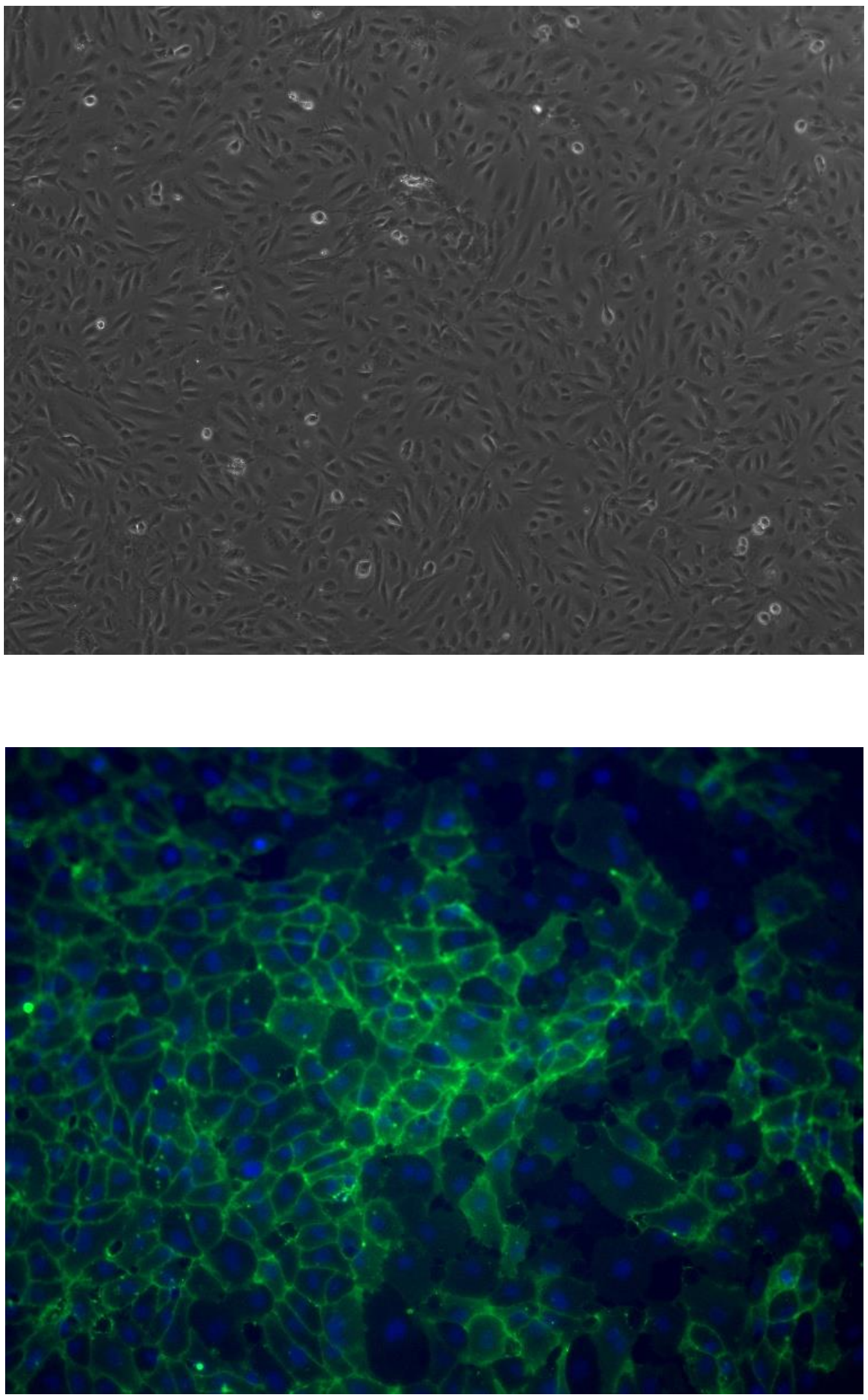

Supplementary Figure S1. Morphological and immunophenotypic characterization of HUVEC cultures. Representative images showing (a) the characteristic cobblestone-like morphology of confluent HUVEC monolayers under phase-contrast microscopy and (b) CD31 immunofluorescence staining. CD31 is shown in green and nuclei are counterstained with DAPI in blue. These features support the endothelial phenotype of the HUVEC cultures used in this study. Images were acquired at different magnifications. HUVECs, human umbilical vein endothelial cells.
